# Davis’ Medical History, Chapter II

**Published:** 1850-07

**Authors:** N. S. Davis

**Affiliations:** Prof. of Physiology and Pathology in Rush Medical College


### THE NORTH-WESTERN
### MEDICAL AND SURGICAL JOURNAL.

Vol. III.] JULY, 1850. [No. 2

### Part 1.—Original Communications.

### CHAPTER 1.—continued.

*History of the Medical Profession from the first settlement of the British Colonies in America, to the year* 1783.—By N. S. Davis, M. D., Prof. of Physiology and Pathology in Rush Medical College.

[Copy Right Secured.]

They were drawn together by suppers in winter, and dinners in summer. Their food was simple and taken chiefly in a solid form. The liquors used with it were Punch, London Porter, and sound old Madeira Wine.

From this general use of distilled and fermented liquors, drunkenness was a common vice in all the different ranks of society."* Who can feel surprised, that in the midst of a people indulging in such habits, death from " Gout, Apoplexy, Palsy, obstructed livers, and Dropsies," should be remarkably frequent?

The same writer tells us that, one club, " consisting of about a dozen of the first gentlemen in the city, all paid, for their intemperance, the forfeit of their lives; and most of them with some one, or more of the diseases that have been mentioned."

*See Rush on the state of Medicine, between 1760, 1766 and 1809.

These few facts are introduced here, for the purpose of showing how poorly we are qualified to judge of the propriety of any system of practice, at a given period of time, without an intimate acquaintance with the condition and customs of the people at the same period. We have already said that practitioners during most of our colonial period, were much controlled in practice by the doctrines of Boerhaave; but during the latter part of the time, these began rapidly to yield to the advancing school of Cullen. There were not wanting, however, many bold and independent practitioners, who did not hesitate to throw off the shackles of prevailing systems, and adapt their treatment to the prevailing type or character of disease, or introduce new remedies, as the preceding facts abundantly show. Of these, none were more deserving of notice than Drs. Boylston, Colden, Ogden, Bayley, Morgan, and Bond. So rapid was the decline of the Boerhaavian influence that before the close of the Revolutionary war, we find blood-letting, Purges, Vomits, Bark, Opium, and Mercury, all used with more or less freedom throughout the profession. The last named remedy was extensively and very successfully used by some physicians, in preparing the system for the small-pox, both in the natural way, and after inoculation.—This practice was commenced as early as 1745, by Drs. Thomas of Va., and Munson of Long Island. And by it, we are told, they reduced the number of deaths after inoculation from 1 in 100, to 1 in 800 or 1000. Dr. Zabdiel Boylston of Boston, first introduced the practice of inoculation for the Small Pox, into the country by inoculating his own son, thirteen years of age, and two colored servants. This was on the 27th of June 1721, only two months after the inoculation of the daughter of the celebrated Lady Wortley Montague, the first that was practiced in England, and certainly before any knowledge of the latter case could have reached Boston. Dr. Boylston was induced to commence the practice by Rev. Dr. Cotton Mather, who had read an account of the inoculation in Turkey, published in the transactions of the Royal Society of London. His three first experiments proving successful, and the Small Pox raging fearfully in the city of Boston, Dr. Boylston, inocculated during that year 247, of all ages and both sexes, and 39 were inoculated by other physicians; making a total of 286, of whom only six died. While of 5,759 who were attacked with it in the natural way, during the same period, 844 died. It is not strange that when this practice was first introduced, it should have met with most violent opposition, requiring no small degree of firmness and perseverance on the part of those who had undertaken it, to stem the torrent of popular indignation which was much exasperated by the conduct of a portion of the Profession, at the head of whom, was Dr. Wm. Douglass, a man of much ability and strong prejudices. Indeed, the history of our Profession, brings to light no controversy of a more exciting and violent character, than that which arose, both in the colonies and the mother country, from the introduction of this practice. And none better illustrates the power of truth, to triumph over error, even when entrenched behind the strongest bulwarks of human prejudice and passion. For the success of Dr. Boylston, rapidly won to the cause intelligent members of the profession, and in a short time, the practice was extended through all the Colonies. And as we have already stated, such was the success attending a proper preparation of the system for the reception of the disease, that, instead of loosing six out of two hundred and eighty-six, as shown during the first year of its introduction, in less than twenty years after, the average mortality of those inoculated did not exceed one in eight hundred. Notwithstanding, the popular prejudice which was excited against Dr. Boylston, was for a time strong and highly embittered, yet he lived to receive ample compensation in the good opinion of his fellow men, both at home and abroad. He afterwards visited England, and besides receiving the most gratifying marks of attention, he had the honor of being the first physician in the Colonies, who was made a member of the Royal Society of London. We are told by Dr. J. W. Francis, in his Anniversary Discourse before the New York Academy of Medicine, that Dr. Beekman Van Beuren, who succeeded his father, John Van Beuren, as physician to the Alms House in the City of New York, "was the first physician who introduced the practice of inoculation for the Small Pox in our public institutions." The first *Post Mortem* examination that took place in America, of which we have any record, was made in 1691, by Dr. Johannes Kerfbyl, assisted by five other physicians of the city of New York. The body examined was that of Governor Sloughter, who had died suddenly under suspicious circumstances. It was not until 1750, nearly sixty years after, that the first human body was dissected, for the purpose of imparting Medical instruction in the Colonies. This was done by the learned Drs. John Bard, and Peter Middleton. The study of Botany also received attention from some of the earliest and most learned physicians of the Colonies.—Among these, Drs. Cadwallader Colden of New York, and John Bartram of Pennsylvania stood pre-eminent. The first named of these eminent men, taught the Linnæan system or classification of plants, on the banks of the Hudson, several years before it was recognized in England. Colden doubtless derived his knowledge from the traveller Kalm, who was a pupil of Linnæas, and so highly was he esteemed by the Botanists of the old world, that one of the most beautiful plants of the tetrandrous class was named Coldenia, in honor of his memory. John Bartram, though living at a little later period of our history, enjoyed a still more extensive reputation in this department of science, and probably, was favored with a more extensive correspondence with the scientific men of Europe than any of his cotemporaries. He was the first Anglo-American who attempted to establish a Botanic Garden in this country. This Garden was located on the Schuylkill, about three miles from Philadelphia, and contained about five acres, well covered with a great variety "of new, beautiful and useful trees, shrubs, and herbaceous plants."

We are told that his garden attracted the attention, and induced the visits of many learned men; and Bartram himself, continued to travel extensively through the country, and make collections in the various departments of natural science, until he was over 70 years of age.

Dr. John Clayton, who came from England to Virginia in 1705, has already been referred to as the author of the *Flora Virginica*, published in 1743; and Dr. Alexander Garden of South Carolina, was no less distingnished both as a naturalist and physician. He kept up a correspondence with Linnæus in Latin, by whom he was held in high estimation. The beautiful flowering shrub called Gardenia, was so named in honor of Dr. Garden. The practice of sending native young men from the colonies to be educated in the Medical Schools of Europe, was more prevalent in the Southern than the Eastern colonies. So early as 1734, William Bull, a native Carolian, received the degree of M. D. at Leyden. John Moultrie graduated at Edinburgh, in 1749, and published his Thesis, "de Febre Flava;" and we are told by Dr. Ramsey, that ten other natives of that colony obtained the same honor between 1768 and 1778. In the middle and southern States, there was much less disposition to merge the duties of Physician and Priest in the same hands, than in New England. Indeed, they seem to have been much better supplied at an early period, with well educated Physicians from England and Scotland, than the more northern colonies; and in the same proportion did tne profession enjoy a higher reputation, both at home and abroad.

East New Jersey appears to be entitled to the credit of making the first attempt, to improve the condition of the profession by means of social organization. So early as 1766, a numerous meeting of physicians was held at New Brunswick, and a constitution and By-laws adopted for a permanent organization. Delegates were present from all parts of the State, and the objects as set forth in the Preamble to the Constitution were, "*Mutual Improvement, the advancement of the Profession, the promotion of the public good, and the cultivation of harmony and friendship among their brethren.*" These were objects worthy the attention of enlightened minds; and they seem to have been strictly adhered to, and successfully carried out; the society meeting regularly twice in each year, until interrupted in 1775, when not a few of its members, left the social circle and the bed side, to bind up the wounds of bleeding freemen, or with them, share the deadly strife in freedom's cause. The second important movement in this direction, was in the colony of Massachusetts, a little before the close of the war. In 1781 the Massachusetts Medical Society was incorporated, embracing about thirty members, with the venerable Edward Holyoke, as its President. Among the original members, we find the names of John Warren, Aaron Dexter, Joseph Fisk, Edward A. Holyoke, and James Lloyd.

During the same year, Dr. John Warren, who was then Surgeon of a Military hospital in Boston, commenced a course of anatomical lectures, which were the first, of which we have any account in New England. They were continued several years, and attended by many of the students of Harvard University, until a Medical Faculty was organized in connection with that institution.

Such is a brief view of Medical institutions and Practice, during the colonial period of our history. And if we consider the condition of the American colonies, the many dependences on the mother country, on the one hand, and the almost constant aggressions of the French and Indians, on the other, the scattered state of the population &c., we doubt whether any profession, under similar circumstances, ever progressed with greater rapidity than the medical profession of the colonies, during the 25 years succeeding the middle of the eighteenth century. Still Quackery in all its forms and guises, was every where flourishing; and the profession itself destitute of that internal organization, or associated effort at improvement, which is so necessary to high respectability and permanent prosperity. Even the prohibitory laws of 1760, in New York, and of 1772, in New Jersey, seem to have had very little influence in accomplishing the object for which they were designed, viz: the elevation of the profession, and the suppression of irregular practice. But the time of their continuance before the commencement of the war of 1775, which for a period of eight years, effectually diverted the attention of all classes from all other subjects, was too short to allow any inference of value, to be drawn in regard to their success or failure. Notwithstanding the adverse circumstances in which the country was placed, and the admitted degradation of a great proportion of the medical practice of those times, still, we cannot but admire the liberal views, the close and patient observation, and the bold cast of originality, exhibited by those whose education had fitted them for the high responsibilities of their calling. And equally must we admire the broad and liberal basis, on which they planted their infant institutions in Philadelphia and New York; requiring as they did, a preliminary education and a curiculum of Medical studies, superior to that of any medical institution now existing in our boasted Republic. If any doubt this, let them compare the regulations of the Philadelphia Medical College of 1768, as already quoted, with those of any of our existing institutions.

### CHAPTER II.

### FROM 1783 TO 1806.

During that great Revolutionary struggle, which terminated in the establishment of the Independence of the American Colonies, but little time or means was afforded for the cultivation of any science or profession, save that of arms and the arts of war. And perhaps no class were more faithful or vigilant in their country's cause, than the practitioners of Medicine. They not onl y followed the Military Camp, sharing its privations and toils as Surgeons, but no small number exchanged the Lancet for the Sword, and the Esculapean wig for the cap of the Military chief. And long will the pages of American history glow with the names of Warren, Mercer, Rush &c., the two former of whom, sealed their devotion to the cause, with their lives. During the war, both New York and Philadelphia fell into the hands of the British, and the operations of the Medical schools, like all other institutions in those places, were suspended until their evacuation. Even the Medical Society of New Jersey, as already stated, was compelled to suspend its functions during the hostile incursions of the proud invader. But while these ordinary means of facilitating Medical education were suspended, another school of quite a different character was opened for the training of our profession. We have alluded in a previous chapter to the benefits derived by the profession of the colonies, from the Medical corps attached to the English armies, sent against the French colonies in the war which terminated in 1763; but the war for Independence afforded another and severer school than the one which had preeeded. For in the former, the colonial profession held the position of pupils and assistants, while in the latter, they were left entirely to their own resources, in a direct contest against their former guides and masters. It was during this trying period, when the profession was compelled to act independently, and often without even a proper supply of drugs, that, animated and encourged by the noble spirit of a Rush, it rose equal to every emergency, and acquired that self-reliance which enabled it, at the close of the eventful struggle, to set about resuscitating its infant institutions, with a zeal that could not fail of success. Hence, no sooner was Philadelphia evacuated by the British army in 1778, than we find the functions of the College and Hospital resumed by the same teachers, and under the same regulations, as before. The number of students in attendance during the winter and spring of 1779, was not less than sixty, although no graduates are reported for that year. Indeed, the number of graduates during the whole of the period under consideration, amounting to near a quarter of a century, scarcely averaged ten annually. This was neither owing to the small number of students in attendance on the Lectures, or to the difficulties in the way of graduation, but to the much less relative importance which was at this period attached to the Degree. The Degree, either of Bachelor of Medicine, or Doctor, conveyed, at hat time, no special privileges in regard to practice; and hence was regarded, like all other literary degrees, as a mere honorary title.

Therefore, we find Students resorting to the Medical College, not so much for the title it was authorized to bestow, as for the real Medical knowledge it was capable of dispensing. In 1779, the Pennsylvania Legislature abrogated the charter of the College, and founded another, called the University of Pennsylvania, endowing it liberally with funds derived from the confiscated estates of the royalists of that commonwealth, and providing for a Medical department in place of that in connection with the one whose organization had been dissolved.

This, however, produced but little other effect on the Medical College than a change of name; for the same professors were in reality continued until 1789, when the Legislature, acknowledging the injustice of their predecessors in abrogating the former charter, restored it again, with all the powers and privileges belonging thereto, but at the same time permitting the University to remain unaltered. This act brought into existence two Colleges, each possessed of the privilege of having a Medical department annexed, and neither willing to forego that privilege in favor of the other.

The result was the organization of two medical faculties during the following year. But experience soon taught them that the patronage was too limited to sustain two institutions, either Literary or Medical; hence they were united in 1792, under the name of the University of Pennsylvania, which has been retained unto the present time. The Medical faculty of the united school, was composed of Dr. William Shippen, Prof. of Anatomy, Surgery, and Midwifery; and Dr. Caspar Wistar, Adjunct; Dr. Adam Kuhn, Prof. of Theory and Practice of Medicine; Dr. Benj. Rush, Prof. of the Institutes of Medicine, and clinical Medicine; Dr. James Hutchinson, Prof. of Chemistry; Dr. Samuel P. Griffitts, Prof. of Materia Medica; Dr. Benjamin S. Barton, Prof. of Botany and Natural History. Some important changes were also made, at this time, for the government of the College, and the conferring of Degrees. The Bachelor's degree was wholly abolished, and that of Doctor in Medicine conferred under the following rules, viz:

"1. That the candidate should not be under twenty-one years of age, should have studied Medicine for three years, and for two at least, in connection with the University.—2. That he should have attended, at least, *one* course of lectures of the several professors, with the exception of the professor of Botany and Natural History, and should also have attended one course of Natural and Experimental Philosophy in the Institution, unless he had received instruction in this branch elsewhere; and 3d, that he should undergo a private examination before the faculty, and if approved by them, should be again privately examined by the Faculty in the presence of as many of the trustees as might choose to attend, and finally, if found to be properly qualified, should offer a thesis, to be printed at his own expense, and defended in public at the Annual Medical Commencement."*

No one can fail to mark the contrast between these requisitions, and those adopted by the first College in 1768. Here

*See "an historical sketch of the University of Pennsylvania," published 1836.

instead of a preliminary education, embracing Mathematics, the Natural Sciences, and some knowledge of the Latin Language, we have a simple requirement in regard to some instruction in "Natural and Experimental Philosophy;" and indeed, the whole requisitions for the Degree of Doctor of Medicine, are much below those previously required for that of Bachelor. And we may here date the commencement of that lowering policy, and that disregard of preliminary education on the part of Medical schools, which has worked great injury to the profession. But why was the original standard departed from, and particularly in the downward direction? Before answering this question, we will turn our attention to the condition of Medicine in the neighboring city of New York.

Soon after the close of the war, attempts were made to revive the Medical department of Kings (now changed to that of Columbia) College. Through some mismanagement the attempt not only failed, but was attended with some circumstances which gave rise to a strong popular outbreak, commonly called the "Doctor's Mob." This arose from a suspicion that some bodies had been stolen from the grave yard for dissection. The mob broke into the dissecting room of the College, and finding several subjects partially dissected they exhibited the fragments to the multitude without, which so increased the excitement, that all law and order were trampled under foot for two or three days. Several medical gentlemen were grossly insulted, and many of the students were confined in prison for personal safety. This unhappy event, not only tended very much to degrade the profession in the public estimation, but also, greatly to retard the progress of the College.

To counteract, as far as possible, the evil influences brought to bear on the profession, and to improve Medical Science, several of the more enlightened young members formed themselves into a private Society. And in 1787 they succeeded in inducing the Magistrates of the City to establish an Apothecary's shop at the public expense, and freely tendered their professional services for the sick poor. Among the more prominent of these men, were Drs. Wm. Moore, Nicholas Romayne, Benjamin Kissam, Wright Post, and Valentine Seaman. They not only gave gratuitous attendance on the poor, for several years, but connected therewith Lectures on most of the branches of Medicine, thereby constituting the first real Hospital and Dispensary, connected with practical instruction, under the corporation of the City. So great was their success that in 1790, more than fifty students attended their instructions. Encouraged by their success, and failing in the establishment of a College of Physicians and Surgeons for the sole purpose of Medical instruction, another effort was made to revive the medical department of Columbia College. In the fall of 1791, the private association introduced no less than sixty medical students into the College, and thereby induced the Legislature to make a grant of over $30,000 to the trustees, for the purpose of enabling them to enlarge their buildings &c. In the following year the Medical faculty was re-organized by the appointment of Drs. Baily, Post, Rogers, Hamersly, Nicoll, and Kissam, Professors, and Dr. Bard, Dean of the faculty. Some of these appointments were so unsatisfactory to the students that many of them abandoned the College, and erased their names from its register. Indeed, such were the internal jealousies, and outward prejudices, that the institution, though it maintained an existence until 1810, yet never attained a degree of prosperity equal to the private association to which we have alluded. About this time also, the buildings for the New York Hospital, which had been destroyed by fire previous to the war, were again so far completed as to allow of the admission of patients. Soon after the close of the war, the subject of Medical instruction began to attract attention in the Eastern States. And we have already seen that so early as 1782, some courses of Lectures on the different branches of Medicine were given, in connection with Cambridge University. This was mainly in consequence of several liberal donations, from some wealthy and enlightened friends of the cause. In 1788, a Medical faculty was established in connection with Havard College, headed by Dr. John Warren, with Drs. Waterhouse, and Dexter for associates. This was continued with a reasonable degree of success, and under fair regulations until 1810, when it was removed to Boston, where it soon obtained a much higher degree of prosperity. How much, the almost simultaneous establishment of these several schools, and particularly the school and Hospital in the rival city of New York, influenced the marked lowering down of the requisitions for graduation, to which we have alluded, in the Medical School of Philadelphia, every reader must judge for himself. It was doubtless these things, aided by the prevailing spirit of the times, that led to this sad change of policy.

The Massachnsetts Medical Society was established by an act of the Legislature of that State in 1781. The objects set forth in the act of incorporation were, the promotion of Medical science and the regulation of all matters pertaining to the profession. To enable it to accomplish these desirable objects, the society was authorized to appoint a Board of Censors, whose duty it was to examine all candidates for admission into the profession in that State, and grant Licenses to such as were found qualified. This Society, together with that formed soon after in New Haven, and the New Hampshire Society, chartered in 1791, exerted a very salutary influence over the profession throughout the United States. Their powers and duties were modified from time to time, until at length the organization of each became complete, and they had severally adopted fixed regulations for the examination and admission of candidates, and enlightened codes of Medical Ethics, as we shall see in the sequel.

To stimulate into action individual talent, and encourage still further the cultivation of Medical Science, a wealthy and enlightened citizen of Boston, Ward Nicholas Boylston Esq. established In 1798 a perpetual legacy, yielding $133 per annum. Thirty-three dollars of this sum was to aid in the establishment of an anatomical Museum, and the remaining one hundred to be awarded annually, for premiums for Medical Essays under the direction of the Fellows of the Massachusetts Medical Society. The noble intentions of the donor have been faithfully carried out by the Society, who have thereby annually called forth a number of interesting Essays, which now embrace many of the most important topics belonging to Medical Science. The Massachusetts Medical Society, also enjoys the honor of being the first in this country to issue a regular volume of transactions, made up of the most interesting papers read before the Society.

The first number of the transactions was published previous to the year 1800, and contained papers written by Drs. E. A. Holyoke, of Salem, Wm. Baylies, of Dighton; Joseph Orne, of Salem; N. W. Appleton, of Boston; Edward A. Wyer, of Halifax, N. S.; Isaac Rand, of Cambridge; Isaac Rand Jr., of Boston; Joseph Osgood, of Andover; Thomas Welsh, of Boston; and Thomas Kast, of Boston. The most important of these papers, were: "An account of the weather and epidemics of Salem in the county of Essex, for the year 1786; with a bill of mortality for the same year. By Edward A. Holyoke, M. D. written in 1787." "A case of Empyema, successfully treated by an operation, by Isaac Rand in 1783." "Observations on Hydrocephalus Internus, by operation, by Isaac Rand Jr., in 1789;" and "an account of an Aneurism of the thigh perfectly cured by an operation, and the use of the limb preserved, by Thomas Kast, in 1790." The second number of the Society's tranactions was not published until 1808. The first Medical Society organized south of New Jersey, was the Philadelphia College of Physicians, which was instituted in 1787, and incorporated by the Legislature of the State in 1789. The Philadelphia Medical Society was organized in the same City, in 1789, and was incorporated in 1792. An interesting notice of the history of the last named Society, may be found in the Medical News and Library for January 1843.

It was during the period under consideration, that another powerful means of diffusing Medical knowledge, and promoting the welfare of the profession, was brought into requisition. I allude to the Medical Periodical Press. The first Medical Periodical published in America, was commenced in the city of New York, and was called the Medical Repository. It was commenced by Drs. Samuel L. Mitchell, Edward Miller, and Elihu H. Smith, 1797; and the first named of this eminent trio, continued its principal editor through the whole of the first sixteen volumes. At the end of this time it passed under the editorial care of Dr. James R. Manley, assisted by able associates, who maintained its reputation unabated until the end of the twenty-third volume. The Repository was a good sized quarterly Journal, and its pages were enriched with many of the ablest Medical and scientific productions of the period through which it was published. In 1804, two Medical Journals were started in Philadelphia, one called the Philadelphia Medical Museum, the other, the Philadelphia Medical and Physical Journal.

The first number of the Museum was issued in September, 1804, edited by Dr. John Redman Coxe. The Medical and Physical Journal was commenced in November of the same year, under the editorial management of Benj. Smith Barton. These several Journals soon called into action much talent that had hitherto been dormant, by eliciting essays and communications from many of the most intelligent members of the profession in every part of the country, by publishing the proceedings of Medical Societies at home and abroad, and by affording a free channel for dignified scientific discussions.—They thus became powerful auxillaries in the great work of Medical education and advancement.

The Medical department of Dartmouth College was organized in 1797, on much the same plan with the first schools in Philadelphia and New York. During the first nine years after the Medical department was established, Medical honors were conferred on 33 candidates, only one of whom took the higher degree of Doctor of Medicine, all the rest taking the degree of Bachelor. The same course was continued up to 1812, after which, the degree of of Doctor, seems to have been the only one conferred by the College. During the period now under consideration, we find very little done by the several State Legislatures, either to promote the education of the profession, or protect the interests of the community against empiricism. The first Legislative act which we find on record, having for its object the regulation of Medical practice, subsequent to the war; was adopted by the Legislature of New York in March 1792. This law required all students who had graduated at some literary College in the United States, to study two years, and those who had not so graduated, to study three years with some reputable practitioner; and then undergo an examination before the Governor, Chancellor, the judges of the Supreme Court, the Attorney General, the Mayor and Recorder of the City of New York, or any two of them. The examining officers were allowed to take to their assistance any three practitioners whom they might choose; and if the candidate was found qualified, he received a License which authorized him to practice Medicine in all its branches. But all persons destitute of such license, were prohibited from collecting pay for their services, except such as were already in practice before the law was enacted. This law was in all respects similar to the Colonial law of 1760; and like it, was limited in its operations to the City and County of New York. The act continued in force five years, when it was repealed or rather superceded by another passed by the Legislature in 1797; which prohibited all persons from practicing Physic or Surgery in that State without a license from one or more of the officers mentioned in the act of 1792, nnder a penalty of twenty-five dollars for each offence.

